# The application of absorbable interference screws in anterior cruciate ligament reconstruction of the knee joint

**DOI:** 10.1093/rb/rbag211

**Published:** 2026-09-24

**Authors:** Junfeng Li, Jingying Dai, Zhixiong Lin, He Yang, Daimei Li, Xiaoyan Chen, Xinfeng Wang, Jingru Du, Honglian Dai

**Affiliations:** State Key Laboratory of Advanced Technology for Materials Synthesis and Processing, Hubei Key Discipline Laboratory of Orthopedic Tissue Injury and Repair, Wuhan University of Technology, Wuhan 430070, China; Department of Orthopaedics, The Third Hospital of Zhangzhou, Zhangzhou 363003, China; State Key Laboratory of Advanced Technology for Materials Synthesis and Processing, Hubei Key Discipline Laboratory of Orthopedic Tissue Injury and Repair, Wuhan University of Technology, Wuhan 430070, China; Department of Orthopaedics, The Third Hospital of Zhangzhou, Zhangzhou 363003, China; State Key Laboratory of Advanced Technology for Materials Synthesis and Processing, Hubei Key Discipline Laboratory of Orthopedic Tissue Injury and Repair, Wuhan University of Technology, Wuhan 430070, China; Key Laboratory of Carbon Fiber Development and Application, School of Chemistry and Life Sciences, Changchun University of Technology, Changchun 130012, China; Key Laboratory of Carbon Fiber Development and Application, School of Chemistry and Life Sciences, Changchun University of Technology, Changchun 130012, China; Department of Orthopaedics, The Third Hospital of Zhangzhou, Zhangzhou 363003, China; Department of Cardiology, The Second Affiliated Hospital of Fujian Medical University, Quanzhou 362000, China; State Key Laboratory of Advanced Technology for Materials Synthesis and Processing, Hubei Key Discipline Laboratory of Orthopedic Tissue Injury and Repair, Wuhan University of Technology, Wuhan 430070, China

**Keywords:** β-TCP/PLGA, bioabsorbable interference screw, ACL reconstruction, osseointegration, biodegradation

## Abstract

Anterior cruciate ligament (ACL) injury is a common sports-related condition that can result in knee instability, pain, functional impairment and subsequent osteoarthritis. ACL reconstruction relies on reliable graft fixation, for which conventional metallic and bioabsorbable interference screws have limitations in biocompatibility, degradation behavior or mechanical performance. Here, we developed a bioabsorbable interference screw composed of 30% β-tricalcium phosphate (β-TCP) and 70% poly(lactic-co-glycolic acid) (PLGA) for ACL reconstruction. The mechanical properties, *in vitro* degradation, biocompatibility and bone–implant integration of the screw were systematically evaluated and compared with those of the commercially available MILAGRO screw in an ovine ACL reconstruction model. The experimental screw exhibited adequate initial mechanical stability, gradual degradation, favorable biocompatibility and progressive bone ingrowth at the screw–bone interface. Its overall degradation and tissue integration profiles were comparable to those of the commercial control. In addition, a clinical study involving 64 patients demonstrated favorable functional outcomes at 6 months after ACL reconstruction, with no major implant-related adverse events observed. Collectively, these findings support the potential of the β-TCP/PLGA interference screw as a bioabsorbable fixation device for ACL reconstruction, combining mechanical stability with progressive degradation and bone integration.

## Graphical Abstract

**Graphical Abstract:**
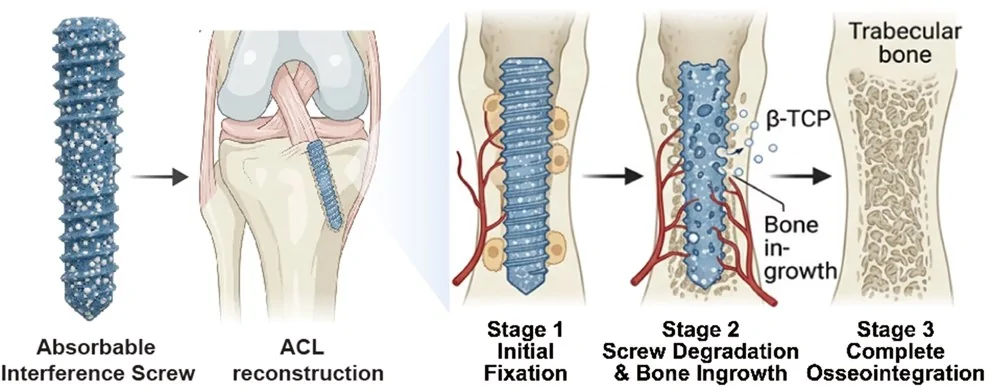

## Introduction

The anterior cruciate ligament (ACL), connecting the femur and tibia, is critical for the stability and functionality of the knee joint [1]. ACL injuries are prevalent in athletic activities, with an annual incidence of ∼200 000 cases in the USA, often resulting in joint instability, pain, functional deficits and osteoarthritis [2]. The current therapeutic standard for ACL rupture is reconstructive surgery, utilizing either autografts or allografts to substitute the impaired ligament [3].

The efficacy of ACL reconstruction is highly dependent on the secure fixation of the graft within the osseous channels [4]. Common fixation devices, such as metal interference screws, cross pins and endobuttons, are employed; however, these materials are not devoid of complications. Metallic fixatives may lead to the release of ions, promote heterotopic ossification and potentially necessitate subsequent surgical interventions. Biodegradable fixatives are associated with risks such as graft displacement, tunnel enlargement and bone loss [5]. Addressing these limitations necessitates the innovation of a resorbable interference screw capable of providing adequate initial stabilization, promoting progressive degradation aligned with tissue regeneration and supporting neo-osteogenesis and soft tissue growth—an imperative advancement for ACL repair surgery.

β-Tricalcium phosphate (β-TCP) and poly(lactic-co-glycolic acid) (PLGA) are widely recognized as bioabsorbable materials favored for their biocompatibility and degradation profiles [6, 7]. β-TCP, a bioactive ceramic, exhibits commendable osteoconductivity and biodegradability, whereas PLGA, a synthetic copolymer, is known for its substantial mechanical properties and biocompatibility. Each, however, presents challenges when utilized in isolation: β-TCP is mechanically inferior, and PLGA degrades at a languid pace, which could potentially lead to localized acidosis or immune responses [8]. To mitigate these issues, the integration of β-TCP and PLGA into composite materials has been explored to balance mechanical robustness with biological functionality. Composite fabrication can be achieved through two primary methods: embedding β-TCP particles within a PLGA matrix to create particle-reinforced composites or amalgamating β-TCP and PLGA fibers to produce fiber-reinforced composites [9]. Particle-reinforced composites have the advantage of tunable β-TCP content and size, which directly influences the composite’s mechanical strength, degradation rate and osteogenic capacity. A significant limitation, however, is the challenge of achieving homogeneous β-TCP dispersion within the PLGA matrix, which can compromise interfacial compatibility and increase the composite’s fragility [10]. Conversely, fiber-reinforced composites are advantageous for their enhanced ductility and resilience. With advanced spinning techniques, the orientation and distribution of fibers can be tailored to emulate the hierarchical structure of bone. The primary drawback of this approach is the insufficient bond strength between fibers, raising the potential for composite delamination or fiber debonding.

The efficacy of absorbable interference screws fabricated from β-TCP and PLGA for ACL reconstruction is gauged by four key parameters: biodegradability, biomechanical properties, biocompatibility and osteogenicity [11]. Biodegradability denotes the rate and manner in which these screws deteriorate structurally and functionally within the biological environment, eventually being metabolized or eliminated. Biomechanical properties encompass the resistance of the screws to bodily stress and strain, alongside their capacity to maintain the secure attachment of the graft within the bone tunnel. Biocompatibility is the degree to which the screws are non-toxic and do not elicit immunological responses such as sensitization, carcinogenesis, teratogenesis or other deleterious effects. Osteogenicity describes the propensity of the screws to support new bone growth and facilitate integration with the bone [12]. In the current market, various iterations of absorbable interference screws comprising β-TCP and PLGA are either available or in the pipeline. For instance, Corliber has introduced the HercuRegen™ product line. This line employs a novel manufacturing process that uniformly disperses β-TCP within the PLGA matrix, enhancing the distribution of the material in a controlled manner to augment the composite’s mechanical strength and durability.

This study examines the biocompatibility and osseointegration properties of an innovative absorbable interference screw designed for use in ACL reconstruction (Figure 1). Composed of 30% β-TCP and 70% PLGA, this screw ensures adequate initial fixation and gradually decomposes to facilitate new bone and soft tissue growth. This process contributes to the natural reconstruction and the functional rehabilitation. Additionally, the screw is engineered to modulate the degradation pace of PLGA, with the alkaline degradation products of β-TCP countering the acidic by-products of PLGA degradation, thereby diminishing tissue irritation. The screw is also tailored to bolster the mechanical interlock and biological interaction between the screw and the graft or bone tunnel, enhancing stress transference and accelerating bone tunnel healing. Moreover, it circumvents complications commonly associated with metallic or other biomaterials, such as the release of metal ions, heterotopic ossification, necessity for secondary surgery, graft displacement, bone tunnel enlargement and bone atrophy. The current investigation juxtaposes the absorbable interference screw with commercially available counterparts, focusing on its compliance with clinical mandates and providing novel material choices for ACL reconstruction to reduce postoperative complications.

**Figure 1:**
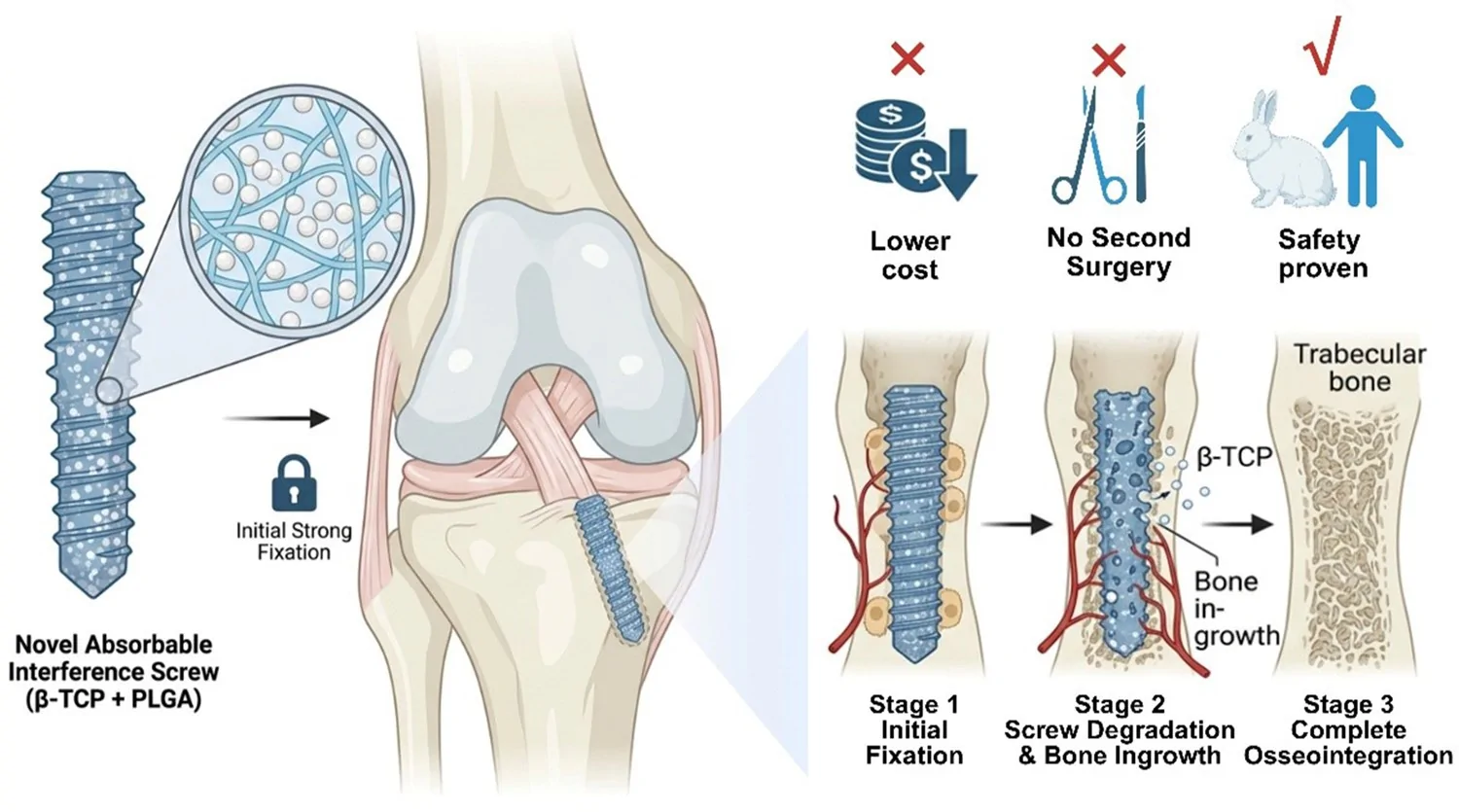
The application of the absorbable interference screw.

## Materials and methods

### Torsional performance testing

To assess torsional performance, the procedure involves clamping the protruding five-thread segment of the interference screw. Given that the screw in question is composed of β-TCP and PLGA—distinct from metallic screws in terms of tolerance to high-load clamping, which could lead to material damage—the method employed here was embedding, with 10-mm exposure, followed by application of a rotational force at a rate of three revolutions per minute until failure. The torque at the point of fracture was subsequently documented. Testing equipment: TTMI02B (Shenzhen Sansi Zongheng Technology Co., Ltd., China).

### Fixed strength mechanical testing

For clinical relevance, tendon ligaments are anchored using fixation screws to compress the graft within the bone tunnel, thus securing the graft. These grafts are typically flattened during compression. In this study, to emulate the bone tunnel, artificial bone block drilling was selected, with suitable surrogate materials chosen to represent the tendon ligament. In accordance with the draft standard ‘Sports Medicine Implant Devices—Tendon Ligament Fixation Screws’, 15 PCF artificial bone blocks were utilized for the tests and nylon rope served as the tendon ligament proxy. Based on comprehensive clinical use and testing methods of competing manufacturers, the testing methods for this study are specified as follows:

#### Fatigue pre-fixed strength test

The fatigue pre-fixed strength test employs a 15 PCF artificial bone block, where pre-drilled holes approximate the bone tunnels. A nylon rope of 3 mm diameter is utilized to represent tendon ligaments. One end of the nylon rope is threaded through the bone block hole, following which the fixation screw is fully inserted into the artificial bone, securing the rope. The base of the bone block is then anchored, and the free end of the nylon rope is subjected to tensile force using the testing equipment (MZ-9000, Xiamen Mingzhi Electronics Co., Ltd., China) at an extension rate of 20 mm/min, with the maximum fixation strength value being recorded.

#### Fatigue performance test

In alignment with the ‘Sports Medical Implants—Tendon Ligament Fixation Screws’ draft standard, the implantation protocol for screws follows the standard. For fatigue performance evaluation, the artificial bone block was secured and tensile stress was applied to the nylon rope using specialized equipment. A cyclic fatigue load ranging from 20 to 200 N is administered for 1000 cycles to determine the extent of slippage of the nylon rope. It is noteworthy that extant literature on knee ligament fixation screw testing reports cycle thresholds below 1000 and cyclic loads not exceeding 250 N.

#### Fatigue fixed strength test

Pursuant to the ‘Sports Medicine Implant Instrument Tendon Ligament Fixation Screw’ standard draft, post-fatigue testing involves recording the maximal fixed strength. This is achieved by stretching the integrated unit of the absorbable interference pin, bone block and nylon rope at a constant rate of 20 mm/min until the screw dislodges or the nylon rope yields (Acumen3- 807.003, Helmstech Co., Ltd., Germany).

### Mechanical testing of rotational torque capability

The ‘Implantable Instruments for Sports Medicine—Screw Fixation of Tendons and Ligaments’ draft standard dictates the measurement of rotational torque (including both screw-in and screw-out torques) for absorbable interference screws. The testing utilized a rigid polyurethane artificial bone with a density of 20 PCF, manufactured by Sawbones, USA, conforming to ASTM F1839 specifications. The rotational torque was tested at a speed of three revolutions per minute. To replicate clinical conditions where screws were fully inserted into bone tunnels, the test screws are also fully engaged.

### *In vitro* degradation test

#### Physicochemical test

Prior to commencing degradation assays, the baseline mass of each group’s absorbable interference screws and sheaths was recorded. The test specimens were subsequently housed in plastic bottles filled with 75 mL of Sorensen buffer solution, sealed and maintained at 37 ± 1°C to ensure full submersion throughout the degradation stages. Monitoring of the buffer solutions’ pH is conducted at defined intervals, with every two weeks assessments across a minimum of two containers. Should any container’s pH deviate from the accepted range, comprehensive pH evaluation across all containers is mandated, with adjustments made to achieve pH 7.4 ± 0.3 utilizing 0.1 mol/L NaOH solution. Turbidity in the buffer solution may suggest microbial contamination and, barring confirmation that such turbidity is attributable to material degradation or its byproducts, the implicated test specimen is to be excluded. The predefined testing intervals include 0, 6, 12, 26, 39, 52, 78 and 104 weeks.

#### Accumulative pH changes

The degradation medium for both the absorbable interference screws and sheaths underwent pH measurement. This was executed by sampling two containers at 4-week intervals for each entity, with the pH values recorded accordingly.

#### Weight loss

Four parallel groups (designated as I, II, III and IV) of absorbable interference screws and sheaths were set up at each stage, and each group of samples was soaked in separate containers. At each prescribed interval, four parallel cohorts (labelled I, II, III and IV) of the absorbable interference screws and sheaths were subjected to degradation in discrete containers. After degradation, samples were vacuum-dried at ambient temperature to a stable mass, following which the degraded specimens’ mass was determined. In the event of fragment generation during degradation, the specimens were cleansed with water of known analytical purity. Subsequently, the specimens and their respective fragments were desiccated to a stable mass and weighed with a balance of 1% precision relative to the total mass. The mass loss percentage is calculated using the formula:


$$(W-W_{t})/W_{0}\times 100\%,$$

where *W*_0_ denotes the initial mass and *W_t_* represents the mass at each respective interval.

#### Infrared spectroscopy measurement

Specimens of absorbable interference screws and sheaths, corresponding to the 0-, 12- and 26-week intervals, were subjected to infrared spectroscopic analysis. Before analysis, these samples were desiccated to a consistent mass. The exterior layers of both screws and sheaths were then dissected into small fragments for infrared spectroscopy utilizing the attenuated total reflectance technique.

#### Calcium ion content measurement

Degradation solutions from the absorbable interference screws and sheaths, pertinent to each defined stage, were centrifuged to extract the degradation fluid. The calcium ion concentration was determined in accordance with GB/T 23942-2009. This involved the preparation of sample solutions, blank solutions and a range of Ca^2+^ standard solutions with concentrations of 0.1, 0.5, 1, 5 and 10 mg/L. Inductively coupled plasma optical emission spectrometry was employed for the analysis. A standard curve was constructed plotting the mass concentration of standard solutions against the corresponding intensity values on the *y*-axis, ensuring a linear correlation coefficient (*R*) exceeding 0.999. The mass concentration of calcium in the sample solutions was then ascertained from the standard curve. Subsequently, the content of calcium ions within the degradation fluid of each parallel sample would be calculated, taking into account the volume of the degradation liquid.

### Mechanical testing after degradation

Artificial bone blocks with a density of 20 PCF were prepared with predefined holes, through which nylon rope was threaded before fully inserting the absorbable interference screws. Each specimen was individually immersed in 800 mL of Sorensen buffer at 37 ± 1°C and maintained under sealed, static conditions. The buffer pH was monitored periodically and maintained at 7.4 ± 0.3 by adjustment with 0.1 mol/L NaOH when necessary. Samples showing evidence of microbial contamination were excluded from analysis. Specimens were evaluated after 0, 6, 12, 26, 39 and 52 weeks of immersion.

Each specimen was used for a single mechanical test to avoid effects from repeated loading. After immersion, specimens were equilibrated at 22 ± 1°C for 10 min. The maximum pull-out force was measured at a constant displacement rate of 300 mm/min. Before testing, the clamped interface surfaces were dried to minimize slippage.

For the baseline (0-week) group, specimens were immersed in Sorensen buffer for 60 ± 3 min and subsequently equilibrated at 22 ± 1°C for 10 min before mechanical testing.

### Animal research

#### Preoperative preparation

A total of 36 skeletally mature male Border Leicester × Merino crossbred sheep (2 years old) were used in this study. Animals were obtained from an approved supplier of the University of New South Wales (UNSW), Australia. All animal procedures were approved by the UNSW Animal Care and Ethics Committee (Ethics Approval No. 16/175A).

The animals were randomly allocated to two groups: the experimental screw group (*n* = 18) and the MILAGRO screw group (*n* = 18). Three animals from each group were assigned to each postoperative evaluation time point (6, 12, 52, 78, 104 and 156 weeks), resulting in *n* = 3 animals per group at each time point.

Animals were housed and cared for in accordance with institutional and applicable animal welfare regulations and were acclimatized before surgery. General health, behavior, mobility, wound healing, food and water intake and signs of pain, distress, infection or other adverse events were monitored throughout the study. All animals underwent preoperative veterinary assessment and were confirmed to be clinically healthy before surgery. Analgesic and supportive treatments were provided when clinically indicated.

Predefined humane endpoints were established in accordance with the approved animal ethics protocol. Animals exhibiting severe or persistent pain, distress or deterioration that could not be adequately alleviated by veterinary intervention were humanely euthanized. Animals with pre-existing conditions or injuries that could affect study outcomes were excluded from experimental procedures and replaced only when necessary to maintain the predefined study design.

#### Screw characterization

For the characterization of the test screw, random samples were imaged using a stereoscopic zoom optical microscope at variable magnifications of 0.67×, 2× and 4× to detail the screw’s microstructure.

#### Collection of autogenous grafts

A precise 0.5-cm incision was made on the dorsolateral aspect of the distal phalanx. The lateral extensor tendon was meticulously separated from adjacent structures, followed by the insertion of a modified Krackow needle at its distal extremity with a size 2 polyester suture. Proximal dissection of the tendon was performed with a ligament stripper, with subsequent transection at the wrist and careful detachment from the fibro-osseous canal. The harvested tendon graft was immediately enveloped in saline-moistened gauze. The operative site was then sutured using 3-0 absorbable sutures (manufactured by Davis & Geck, North Adelaide, Australia) to facilitate closure. Maintenance of graft hydration with saline was imperative during preparation. Subsequently, a Krackow stitch was placed at the alternate extremity of the graft utilizing #2 sutures (Ethibond, Johnson & Johnson, North Adelaide, New South Wales, Australia).

#### ACL reconstruction model surgery

Two-year-old skeletally mature Border Leicester Merino crossbred sheep were used to establish the ovine ACL reconstruction model. Animals were fasted for 48 h before surgery and anesthetized according to the institutional protocol. Under aseptic conditions, the lateral extensor tendon was harvested using a modified Krackow suture technique and maintained in sterile saline-soaked gauze until implantation.

A medial parapatellar approach was used to expose the knee joint. The ACL was completely transected while preserving the posterior cruciate ligament. Femoral and tibial bone tunnels were created according to the anatomical ACL insertion sites using a guide wire and cannulated drill. The harvested graft was passed through the tibial and femoral tunnels and fixed using either the experimental bioabsorbable interference screw or the commercial MILAGRO screw. The tibial and femoral grafts were fixed with ∼40 N of tension. The contralateral left knee received screw implantation at the femoral site only and served as the contralateral comparison.

Following fixation, the joint was thoroughly irrigated with sterile saline and the surgical wound was closed according to standard procedures. Postoperative radiographs were obtained in anteroposterior and lateral views using a portable X-ray system with a digital imaging cassette (AGFA), and images were archived in the Digital Imaging and Communications in Medicine (DICOM) format.

Animals were monitored daily during the first postoperative week and weekly thereafter for general condition, food intake, surgical-site changes, ocular and mucosal status, respiratory and cardiovascular function and locomotor behavior. At the predetermined study endpoints, animals were euthanized with an overdose of Lethobarb. The hind limbs and major organs, including the heart, liver, lungs, kidneys and spleen, were harvested for gross examination and histological evaluation. Regional lymph nodes and abnormal tissues, when present, were also collected and fixed in buffered formalin for further analysis.

#### Radiology

The Faxitron system and an AGFA CR MD4.0 cassette (exposure settings of 32 kV for 25 s) were utilized. The AGFA digital processing software and workstations (AGFACR 75.0 Digitiser Musica, AGFA, Germany) were employed to process the acquired images. The ezDICOM medical viewer was used to convert the DICOM data to JPEG format. Faxitron films of the anteroposterior view were assessed to identify signs of adverse reactions, independently.

#### Micro-computed tomography

Micro-computed tomography (µCT) analyses were conducted employing the Inveon Scanner (Siemens, Germany) to evaluate the reconstructed right knee and the contralateral left knee from the same sheep. The right knee underwent ACL reconstruction with implantation of either the designated test screw or the commercially available MILAGRO screw, according to the experimental group, whereas the contralateral left knee received screw implantation at the femoral site only. Thus, the right and left knees within each sheep constituted paired observations for within-animal comparisons. The scanning protocol was configured for a slice thickness of 50 µm. Images from CT scans were archived in the DICOM format. 3D reconstructions facilitated assessments in axial, sagittal and coronal planes. Evaluations of ligamentous healing, osteointegration and local osseous responses to screw placement were based on the coronal and sagittal reconstructions from the µCT data.

#### Dissection

The ACL graft was carefully dissected from the femoral tunnel and labeled using 5-0 sutures for identification. The ACL was expeditiously excised from the tibial insertion and preserved in formalin for subsequent routine paraffin-embedded histological evaluation. Osteotomy of the femur and tibial tunnel was performed with a margin of 1 cm of the normal cancellous bone retained, inclusive of the left femoral screw site.

#### Paraffin section hematoxylin and eosin staining histology

Tissue specimens were fixed in 10% neutral buffered formalin, routinely dehydrated, cleared in xylene and embedded in paraffin. Sections (5–8 μm) were prepared using a microtome, mounted on glass slides and stained with hematoxylin and eosin (H&E) according to standard histological procedures.

Medial and lateral joint capsule tissues, menisci and intra-articular tissues from both knees were processed for H&E staining to evaluate synovial and meniscal integrity and local tissue responses to surgery or implantation. Articular cartilage specimens from the medial femoral and tibial condyles were fixed in formalin, decalcified with formic acid, paraffin-embedded, sectioned sagittally and stained with H&E to assess cartilage morphology. Heart, liver, lungs, kidneys and spleen were also collected, fixed in formalin and processed for H&E histological examination to evaluate potential systemic pathological responses.

#### Methyl methacrylate histological examination

Specimens were fixed in chilled phosphate-buffered formalin for at least 96 h, with two changes of fixative. Bones containing the bone tunnels and adjacent tissues were sectioned, dehydrated in ethanol and embedded in methyl methacrylate (MMA). Longitudinal sections perpendicular to the bone tunnel axis were prepared at ∼15-μm thickness using a SP1600 microtome (Leica, Germany).

The sections were etched with acidic ethanol (98 mL of 96% ethanol and 2 mL of 37% HCl) and sequentially stained with 1% methylene blue in borax buffer (pH 8.5) and 0.3% basic fuchsin. The bone–graft–screw interfaces of the right tibia and femur were examined to assess bone responses, tissue integration and potential pathological changes. The femoral screw–bone interface of the contralateral left knee was evaluated using the same MMA histological procedure.

#### Histomorphometric analysis

Histomorphometric analysis was performed on PMMA-embedded sections to quantitatively evaluate the tissue composition surrounding the implanted interference screw. A validated customized MATLAB-based SORL analysis program was used for image segmentation and quantitative analysis. The areas occupied by residual interference screw, bone tissue (including newly formed bone and bone marrow) and other soft tissues were identified and quantified. The area percentage of each tissue component within the evaluated region was calculated to quantitatively characterize implant resorption and tissue/bone replacement.

### Clinical trials

To ascertain the efficacy and safety of bioabsorbable interference screws in securing soft tissue or bone–tendon–bone grafts during orthopedic procedures, a verification was conducted utilizing a single-group target value approach and a multicenter clinical study. The study enrolled 64 participants and assessed their knee joint function and mobility pre-operatively and post-operatively at 1, 3, 6 and 12 months, utilizing the Lysholm Knee Scoring System. Additionally, the Lachman test was employed to evaluate the integrity of the ACL. The Lysholm scoring is categorized as follows: 95–100 points is rated as excellent, 85–94 points as good, 65–84 points as fair and any score below 65 points as poor. Concurrently, the study monitored safety-related outcomes by recording the frequency of adverse events, severe adverse events and product defects. The clinical trial was reviewed by the Medical Ethics Committee of the Third Hospital of Hebei Medical University (Ethics Review Reference No. Qi2021-003-1).

#### Patient eligibility

**Inclusion criteria** were as follows: (i) age 18–70 years, (ii) BMI 18–30 kg/m², (iii) meeting the predefined diagnostic criteria and surgical indications, with planned fixation of a soft-tissue or bone–patellar tendon–bone (BPTB) graft using the bioabsorbable interference screw system and (iv) provision of written informed consent after understanding the study requirements.

**Exclusion criteria included** (i) active systemic or local infection or osteomyelitis, (ii) bone defects or severe surrounding soft-tissue injury, (iii) inability to understand or comply with study requirements, (iv) pregnancy or breastfeeding, (v) participation in another clinical trial with incomplete follow-up, (vi) concomitant conditions that could affect study participation, follow-up or data validity, (vii) investigator-determined contraindications or other conditions rendering the patient unsuitable for enrollment and (viii) severe hepatic or renal dysfunction, defined as Alanine Aminotransferase (ALT) and/or Aspartate Aminotransferase (AST) ≥2 × the upper limit of normal (ULN), with serum creatinine and BUN both ≥2 × ULN.

#### Sample size calculation

A literature search was conducted to evaluate the clinical safety and effectiveness of comparable bioabsorbable interference screws. CNKI, Wanfang Data and VIP databases were searched without publication-date restrictions using the terms ‘ligament’, ‘screw’, ‘Lysholm’ and ‘knee joint’. Eligible studies included randomized or non-randomized controlled studies and retrospective case series reporting clinical outcomes, indications, safety or effectiveness. *In vitro* studies, animal studies, reviews, meta-analyses and studies irrelevant to the research objectives were excluded.

The literature search identified an 87.7% rate of good or excellent clinical outcomes for comparable products. The lower bound of the corresponding two-sided 95% CI (84.3%) was adopted as the target value (*P*₀). Based on investigator expectations and prior knowledge of the investigational device, the expected rate of good or excellent outcomes at 6 months postoperatively was set at 96% (*P*₁).

The sample size was estimated using the approximate formula for the single-arm target-value method recommended in the Guideline for the Design of Clinical Trials of Medical Devices issued by the former China Food and Drug Administration. The sample size was calculated using the following formula:


$$N=\frac{{\left[Z_{1-\alpha /2}\sqrt{P_{0}(1-P_{0})}+Z_{1-\beta }\sqrt{P(1-P)}\right]}^{2}}{{\left(P-P_{0}\right)}^{2}}$$

Among these, α = 0.05, β = 0.2, *Z*_1-α/2_ = 1.96, *Z*_1-β_ = 0.842.

Considering an anticipated dropout rate of ∼10%, the final planned sample size was set at 64 participants.

#### Description and analysis of baseline characteristics

The demographic characteristics included age, sex, height and body weight. The participants ranged in age from 18.80 to 68.40 years, with a mean age of 34.07 ± 10.27 years. There were 45 male participants (71.43%) and 18 female participants (28.57%). The mean height and body weight were 173.04 ± 8.59 cm and 76.18 ± 11.23 kg, respectively (Table S1).

Baseline functional status was assessed using the Lysholm Knee Scoring Scale (Tables S2 and S3). Before surgery, the mean Lysholm score was 64.51 ± 20.32 points, with scores ranging from 9 to 100 points. According to the Lysholm grading criteria, three participants (4.76%) were classified as excellent, 10 (15.87%) as good, 23 (36.51%) as fair and 27 (42.86%) as poor. At baseline, all 63 participants (100%) had ACL injuries, while no participants (0.00%) had posterior cruciate ligament (PCL) injuries. The Lachman test endpoint was classified as firm in 1 participant (1.59%), soft in 47 participants (74.60%) and absent in 15 participants (23.81%). Overall, the Lachman test was positive in 62 participants (98.41%) and negative in 1 participant (1.59%).

### Statistical analysis

Statistical analyses were performed using GraphPad Prism (GraphPad Software, USA). Data are presented as mean ± SD. Two-sided *P* < 0.05 was considered statistically significant. Parametric and nonparametric tests were selected according to data distribution and experimental design, including unpaired or paired Student’s *t*-tests and Mann–Whitney *U* or Wilcoxon signed-rank tests, as appropriate.

For animal and clinical outcomes, paired or independent comparisons were performed as appropriate. The Lysholm scores were analyzed for between- and within-subject differences, while clinical effectiveness rates were compared between groups with the between-group difference and 95% CI reported. The Lachman test outcomes were analyzed using the Pearson’s χ^2^ or Fisher’s exact test, as appropriate.

## Results and discussion

### Chemical characterization and mechanical performance verification

Scanning electron microscopy (SEM) was utilized to analyze the morphological features of the bioabsorbable interference screw, with the images depicted in Figure 2A. To ascertain if the mechanical integrity of the bioabsorbable interference screw aligns with clinical demands, a comparative analysis was conducted with the MILAGRO interference screw as a benchmark. This analysis encompassed torsional performance, fixation strength and rotational torque. Torsional resistance was evaluated in accordance with the ‘Implantable Tendon and Ligament Fixation Screw for Sports Medicine’ standard, quantifying the torsional moment leading to screw fracture. Outcomes illustrated in Figure 2B demonstrate that the bioabsorbable interference screw exhibits torsional resilience on par with that of MILAGRO, signifying that it satisfies the clinical criteria for torsional performance.

**Figure 2:**
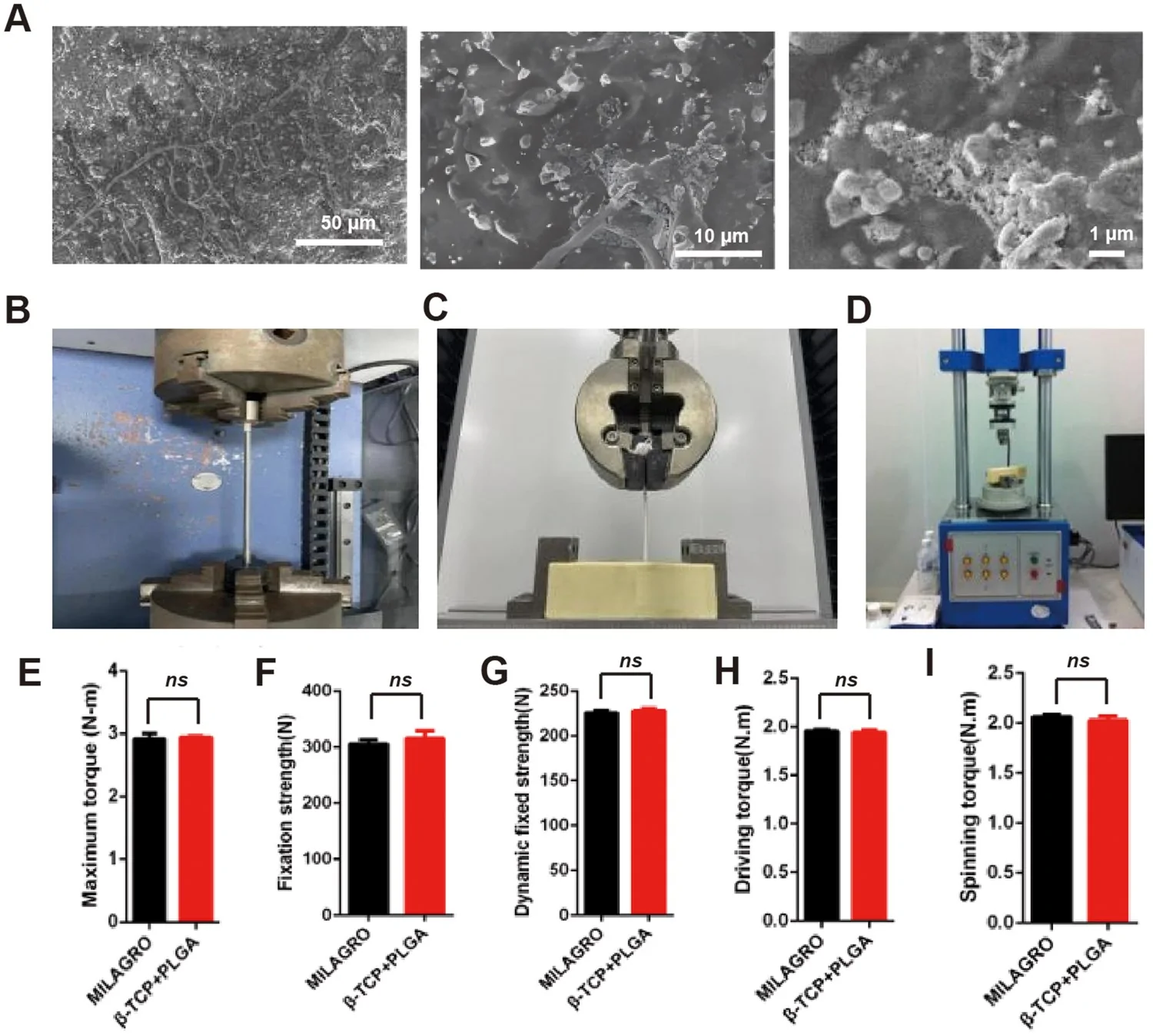
(**A**) SEM imaging of absorbable interference screws at different magnifications. (**B**) Torsional performance testing. (**C**) Static and dynamic maximum fixation strength testing. (**D**) Insertion torque and removal torque testing. The maximum torque (**E**), fixation strength (**F**), dynamic fixed strength (**G**), driving torque (**H**) and spinning torque (**I**) of MILAGRO and β-TCP/PLGA bioabsorbable interference screws.

For fixation strength, the testing protocol followed the same sports medicine standard and documented the maximal static and dynamic fixation strengths. As shown in Figure 2C, the bioabsorbable interference screw and MILAGRO exhibited comparable results in both static and dynamic conditions, confirming that the bioabsorbable screw’s fixation strength is within the clinically acceptable range. Rotational torque assessment, inclusive of both insertion and extraction torque, adhered to the specified standard. Results presented in Figure 2D reveal no discernible difference between the bioabsorbable interference screw and MILAGRO, corroborating that the rotational torque performance of the bioabsorbable screw fulfills the clinical benchmarks.

The clinical viability of β-TCP/PLGA interference screws depends on robust mechanical properties (Figure 2E–I). Safe surgical insertion requires high torsional resistance. The experimental screws exhibited maximum, driving and spinning torques entirely comparable to the commercial MILAGRO controls (Figure 2E, H, I). Postoperative graft stability relies on strong tissue anchoring. Both the static and dynamic fixation strengths of the β-TCP/PLGA group perfectly matched the commercial standard (Figure 2F and G). Ultimately, incorporating β-TCP into PLGA does not compromise structural integrity. The novel composite achieves the essential mechanical equivalence required for secure ligament fixation.

### *In vitro* degradation performance

The *in vitro* degradation behavior of the absorbable interference screws and MILAGRO controls was evaluated over a 104-week period. Representative macroscopic images (Figure 3A and B) and quantitative weight loss analysis (Figure 3C and D) revealed a multi-stage degradation profile. During the initial 12 weeks, both screws maintained structural integrity with a slight mass increase due to water absorption and swelling. Beyond 26 weeks, the screws exhibited a transition to accelerated degradation, manifested by mottled surfaces, blurred thread profiles and a significant rise in weight loss rate, which reached 33.6% at 52 weeks. By week 104, both groups had fragmented into powdery residue, confirming full degradability. This morphological transition was corroborated by FTIR spectra (Figure 3F), where the weakening of the ester bond peak (1753 cm⁻¹) and intensification of phosphate group peaks (1042 cm⁻¹) indicated PLGA matrix erosion and β-TCP exposure. Notably, the pH of the medium (Figure 3E) dipped to ∼6.8 during the peak hydrolysis phase (weeks 32–76) but gradually recovered to 7.3 after 84 weeks. This ‘V-shaped’ recovery, synchronized with the cumulative release of Ca^2+^ (Figure 3G), suggests that the dissolution of β-TCP provided an effective self-buffering mechanism to neutralize the acidic byproducts of PLGA. Mechanically, the pull-out resistance (Figure 3H) remained robust (360–430 N) for the first 26 weeks, sufficient to ensure primary stability during the critical early phase of tendon-to-bone healing in ACL reconstruction. The subsequent decline in mechanical strength after 52 weeks aligns with the biological bone remodeling cycle, where the load is gradually transferred from the implant to the regenerating host tissue. Collectively, these results demonstrate that the developed interference screw possesses a degradation kinetic and mechanical stability profile comparable to clinical standards, with good acid-neutralizing capacity.

**Figure 3:**
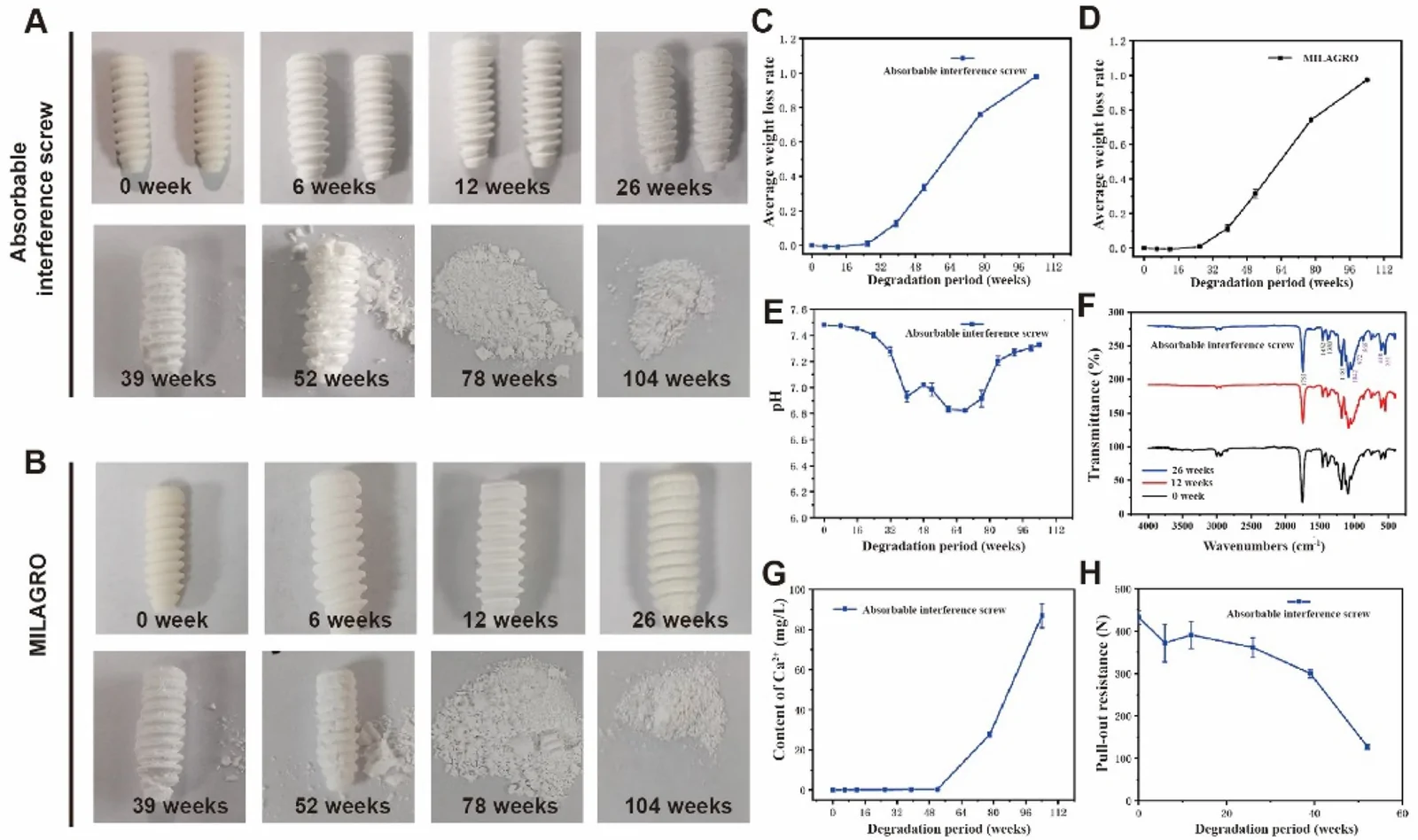
*In vitro* degradation and mechanical performance of the interference screws. Morphological changes of the absorbable interference screw (**A**) and MILAGRO screw (**B**) over 104 weeks. Average weight loss rates of the absorbable interference screw (**C**) and MILAGRO screw (**D**). (**E**) pH changes in the degradation medium for the absorbable screw. (**F**) FTIR spectra showing chemical changes at 0, 12, and 26 weeks. (**G**) Cumulative calcium ion release over time. (**H**) Pull-out resistance of the absorbable screw during 52 weeks of degradation.

### Application of absorbable interference screws in ACL reconstruction of the knee

The study examined the utilization of bioabsorbable interference screws for graft fixation in ACL reconstruction within ovine models (Figure S1A). Post-surgical observations were conducted following euthanasia at 6, 12, 52, 78, 104 and 156 weeks post-reconstruction (Figure S1B). Systematic dissections were performed to assess each specimen for any signs of adverse reactions or infection at the surgical locus. In addition, critical organs including the heart, liver, lungs, kidneys and spleen were harvested for pathological examination to ascertain whether the implant had disseminated any infection to these distant anatomical sites. The outcomes of these investigations are delineated in Figure S1C. Observational data at 6, 12, 52, 78, 104 and 156 weeks postoperative indicated an absence of adverse reactions at the incision site and in the subcutaneous tissue. Furthermore, the condition of distant organs remained unchanged in all specimens, leading to the conclusion that the deployment of bioabsorbable interference screws does not precipitate detrimental systemic effects.

Imaging of knee joint samples from both the experimental and control cohorts was performed using a Faxitron radiography unit (calibrated at 32 kV for a 25-s exposure). The radiographic images, capturing both anterior–posterior and lateral views of the bioabsorbable and MILAGRO screws, were graded in a double-blind evaluation. This process was intended to detect any adverse osseous reactions at the site of screw implantation. According to the findings presented in Figure 4A, there was no evidence of adverse bone responses in either the anterior–posterior or lateral views of the bioabsorbable interference screws at 6, 12 and 52 weeks post-implantation. These findings align with those observed for the commercially available MILAGRO screws. Nevertheless, at the 52-week mark post-implantation, observations included bone hypertrophy and meniscal calcification in both the anterior–posterior and lateral images of the bioabsorbable screws within cancellous bone. Current evidence implies that these occurrences are more likely attributable to the animal model employed rather than the specific screw design, as similar patterns were noted in association with MILAGRO screws.

**Figure 4:**
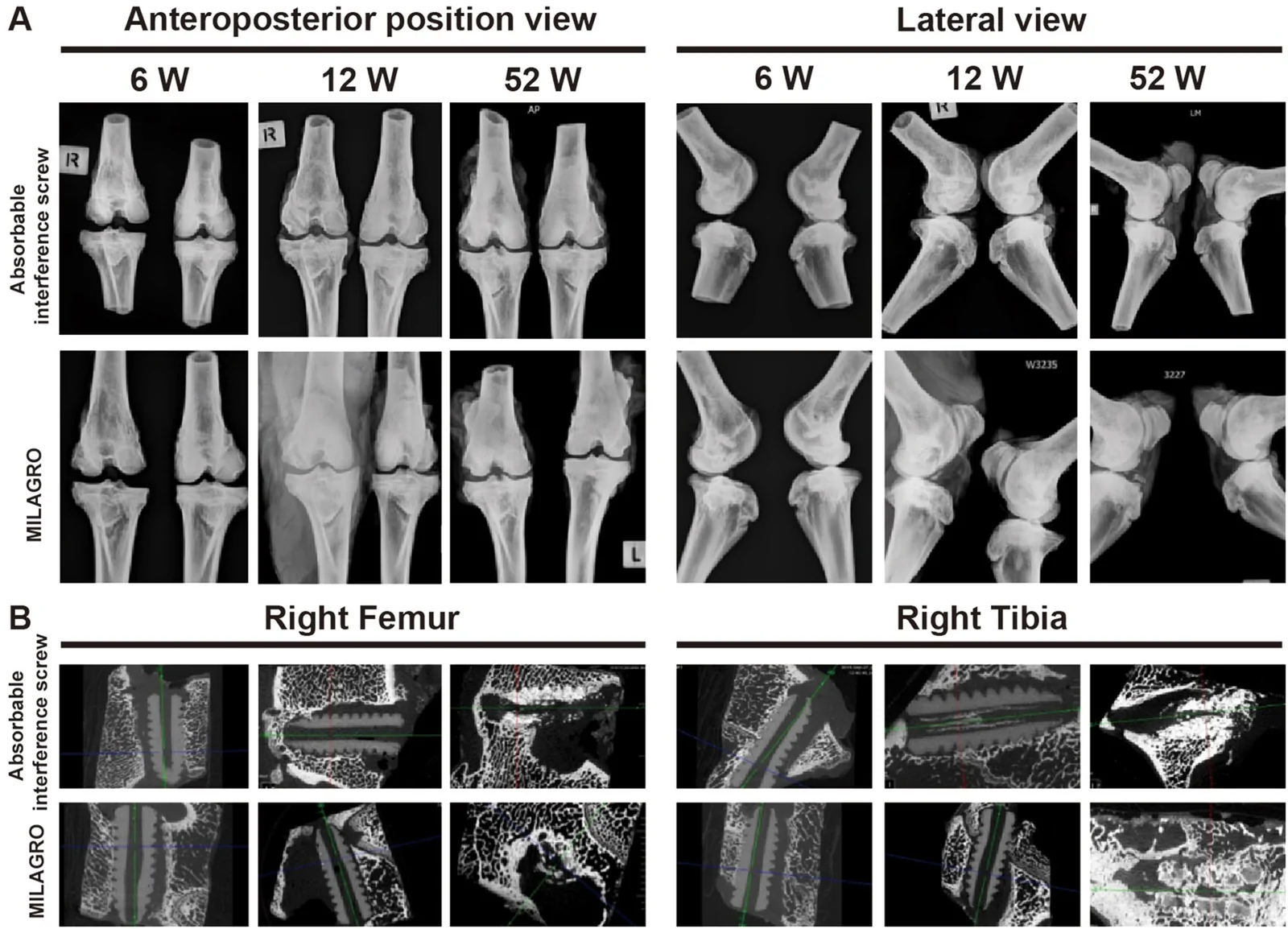
Bone reaction after the implantation of absorbable interference screws. (**A**) The anteroposterior and lateral position view images of absorbable interference screws and MILAGRO. (**B**) Absorbable interference screws and MILAGRO at the tibia and femur in the right knee after ACL reconstruction at 6, 12 and 52 weeks and images of screw integration and degradation.

µCT was utilized, employing the Inveon scanner, to analyze the reconstructed right knee and the contralateral left knee. The resolution of the scans was uniformly set at ∼50 µm. All scans were archived in the DICOM format. Subsequently, 3D reconstructions were generated for detailed examination in axial, sagittal and coronal orientations to assess tendon ossification, screw integration and local osseous response. Micro-CT of the ovine right knee (encompassing the distal femur and proximal tibia) and the contralateral left knee yielded results, as depicted in Figure 4B. Observations at 6 and 12 weeks post-operatively indicated intact bioabsorbable interference screws and MILAGRO screws with only marginal indications of material degradation evident on imaging. Notably, at these intervals, both screw types exhibited pronounced osteogenesis along their thread contours. At the 52-week time point, both the bioabsorbable interference screws and MILAGRO screws were observed to be assimilated within the bone tunnels, with no discernible deleterious responses. Concomitantly, at this same juncture, instances where bioabsorbable interference screws and MILAGRO screws had been implemented in the distal femur and proximal tibia for ACL reconstruction, there was evidence of resorption and fracture of the screws in these locations.

In summary, the bioabsorbable interference screws demonstrated consistent osteogenic activity, resorption and structural integrity consistent with clinical expectations at the sites of the distal femur and proximal tibia.

To elucidate the influence of bioabsorbable interference screws on synovial tissues, specimens of knee joint capsule, articular cartilage and meniscus were procured for histological examination. These tissues were embedded in paraffin, sectioned and subjected to H&E staining. The outcomes, presented in Figure 5A–C, revealed no detrimental reactions in the joint capsule, articular cartilage or meniscal tissues in both experimental and control groups across all evaluated time points, signifying negligible impact of the bioabsorbable interference screws on the synovial structures.

**Figure 5:**
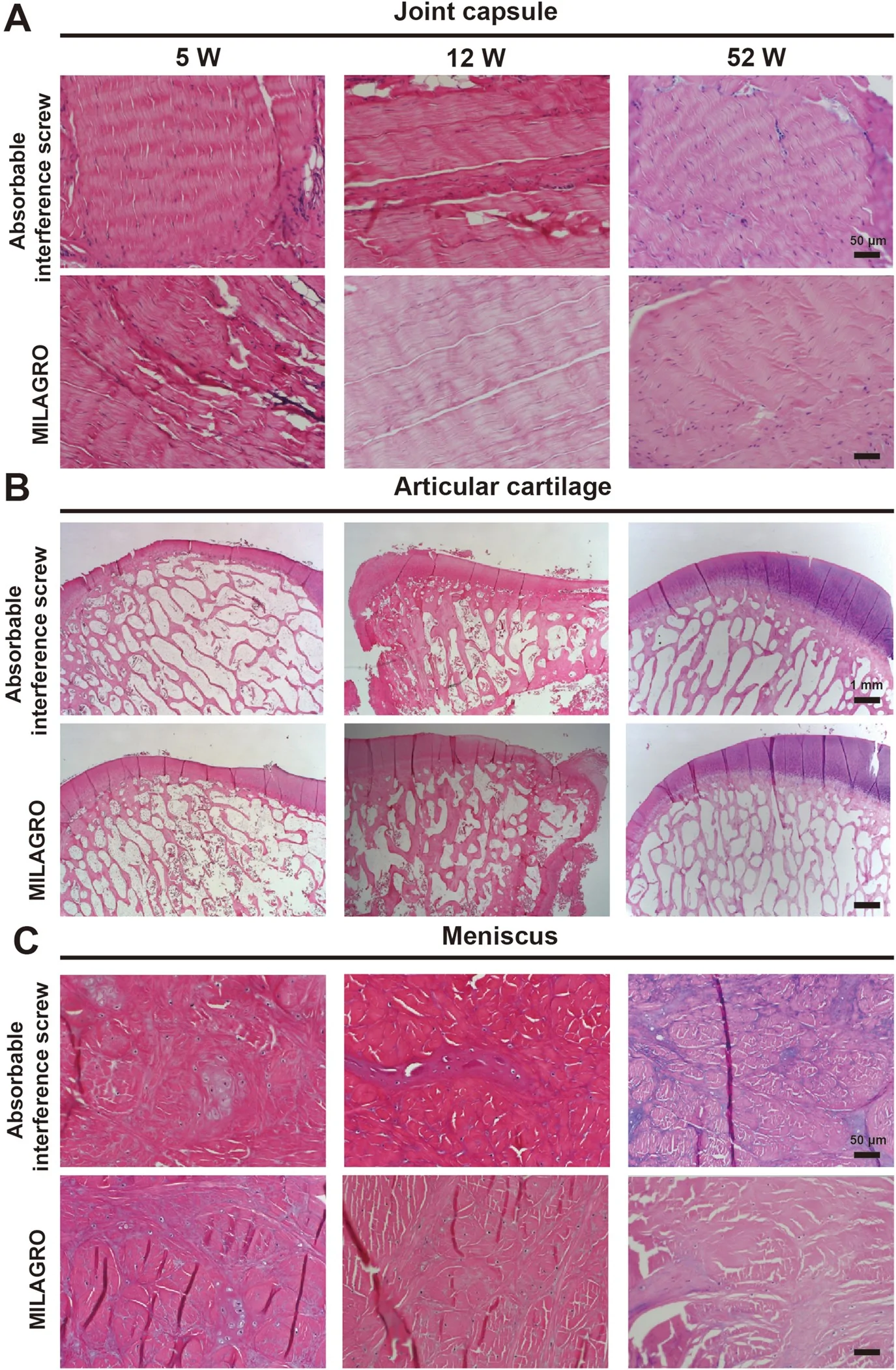
H&E staining of absorbable interference screws and MILAGRO. (**A**) Different time points joint capsule tissue H&E staining image. (**B**) Different time points articular cartilage tissue H&E staining image. (**C**) Different time points meniscus tissue H&E staining image.

The findings indicated a minimal presence of macrophages and a dispersion of lymphocyte infiltration during the implantation period; however, multinucleated giant cells and plasma cells were not detected. Both the experimental and control groups exhibited slight fibrovascular infiltration and evidence of neovascularization at all assessed time points, with a more pronounced occurrence in the initial stages post-implantation. No distinction in cell types between the experimental and control screws was observed at any time point. These observations suggest that bioabsorbable interference screws are biocompatible with synovial tissues and exert an equivalent influence on bone post-implantation as compared to commercially available screws, underscoring their clinical utility.

To evaluate the *in vivo* osseointegration and biocompatibility of the implants over time, histological analysis of the bone-implant interfaces in the femur and tibia was conducted using MMA staining at 6, 12 and 52 weeks post-implantation (Figure 6). At the early stage of 6 weeks, preliminary woven bone formation and initial tissue ingrowth were observed adjacent to the implant surfaces in both the novel absorbable interference screw and the commercial MILAGRO control groups. As the healing progressed to 12 weeks, increased direct bone-to-implant contact and progressive bone maturation were evident across all anatomical sites (left/right femur and tibia). Remarkably, by 52 weeks, extensive active bone remodeling and robust osteogenesis were observed. As indicated by the arrows, mature new bone tissue grew directly onto and intimately interlocked with the surfaces of the implanted screws, demonstrating excellent osteoconductivity and biocompatibility without eliciting severe inflammatory responses. Notably, the newly developed absorbable interference screw exhibited a highly comparable osseointegration and degradation profile to the established MILAGRO screw throughout the 52-week healing process. This direct bone apposition confirms that the absorbable interference screw can provide a favorable microenvironment for cellular colonization and continuous physiological bone ingrowth, highlighting its substantial clinical potential for effective graft fixation in sports medicine applications.

**Figure 6:**
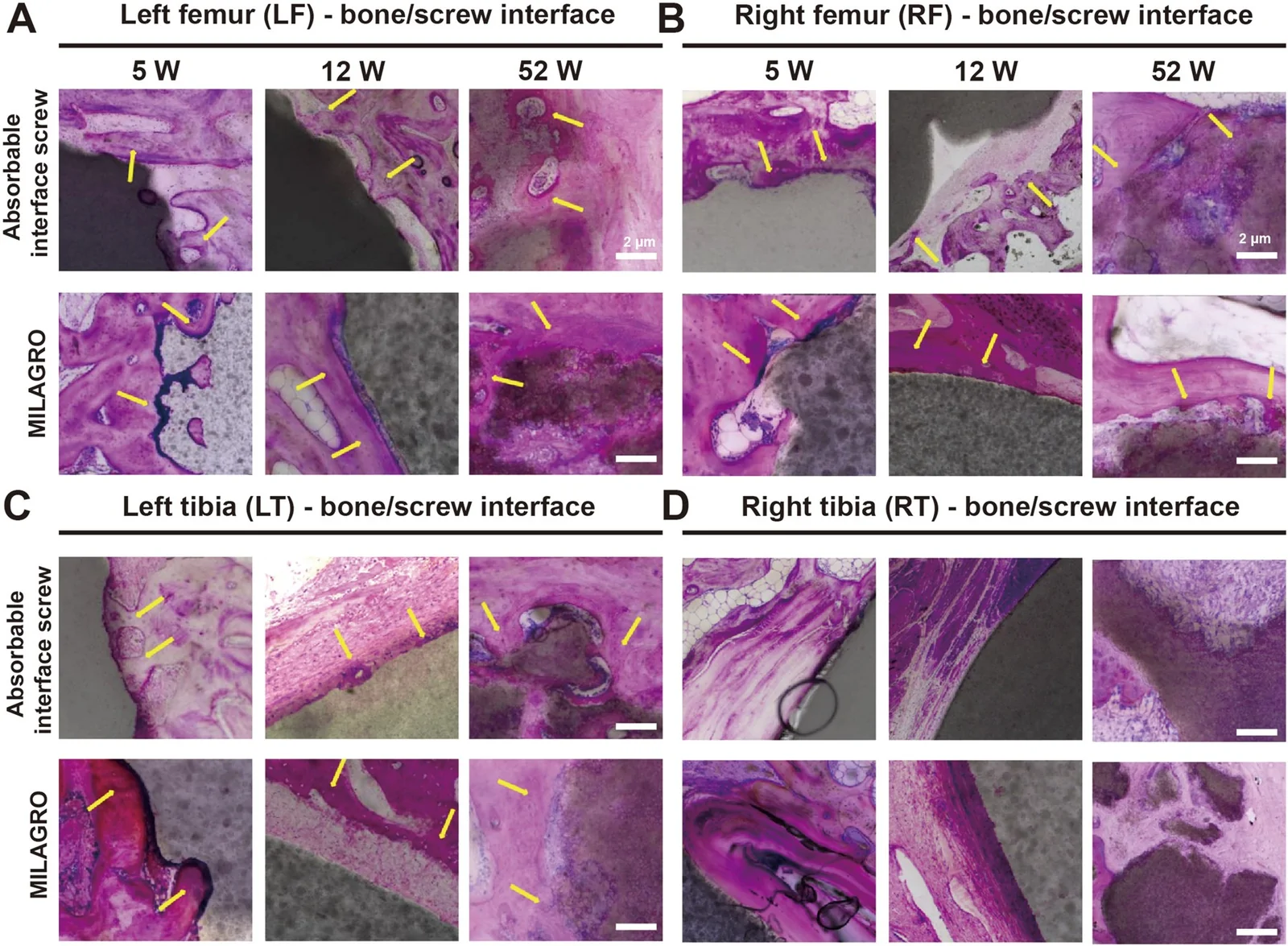
MMA staining of absorbable interference screws and MILAGRO. Different time points screw—bone interface tissue staining images of left femur (LF) (**A**), right femur (RF) (**B**), left tibia (LT) (**C**) and right tibia (RT) (**D**), arrows represent new bone growth directly on the surface of the implanted screw.

To quantitatively evaluate the tissue response and implant degradation, histomorphometric analysis was performed on PMMA sections using a validated customized MATLAB-based SORL program (Figure 7). The area percentages of bone, residual screw, bone marrow and other soft tissues were quantified. As shown in Figure 7, no statistically significant difference in bone area was observed between the test and predicate groups at 6, 12 or 52 weeks (*P* = 0.814, 0.132 and 0.052, respectively). The bone area was therefore comparable between the two groups throughout the evaluated period. The proportion of residual screw material decreased with increasing implantation time, while the proportions of bone marrow and other soft tissues changed accordingly, indicating progressive remodeling of the implant-associated tissue compartment. These quantitative findings were consistent with the representative histological observations.

**Figure 7:**
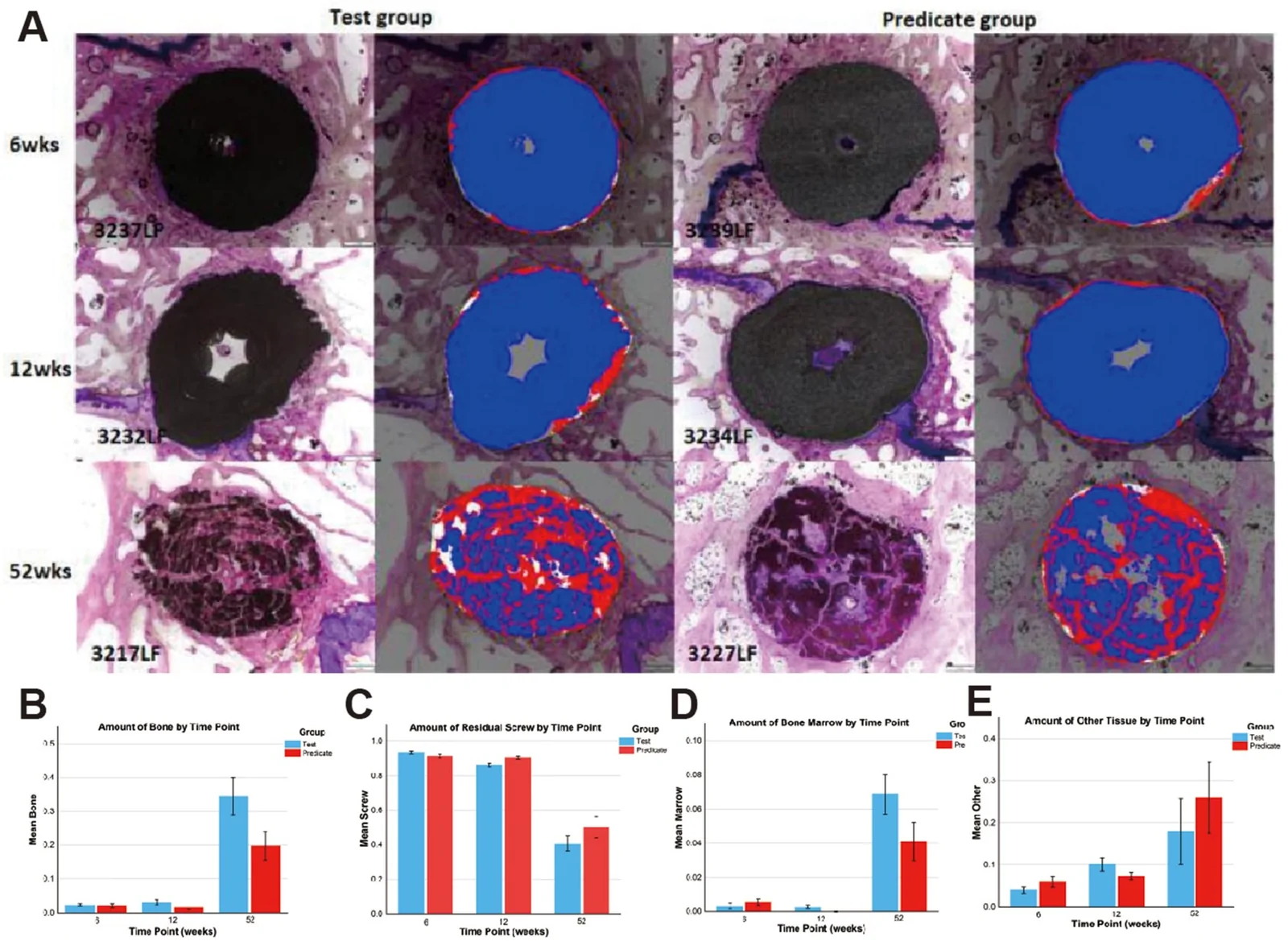
Histomorphometric evaluation of the β-TCP/PLGA interference screw and predicate group. (**A**) Representative PMMA histological sections and corresponding image segmentation at 6, 12 and 52 weeks. (**B**–**E**) Quantitative area percentages of bone, residual screw, bone marrow and other soft tissues, respectively, determined using a validated customized MATLAB-based SORL histomorphometric analysis program. No significant difference in bone area was observed between the test and predicate groups at 6, 12 or 52 weeks (*P* = 0.814, 0.132 and 0.052, respectively).

Building upon the early-stage observations (6–52 weeks), the long-term follow-up from 78 to 156 weeks confirmed the complete resorption of the interference screw and successful bone remodeling. Radiographic analysis (Figure 8A) demonstrated sustained joint stability without evidence of screw migration or joint space narrowing, even as the implant underwent terminal degradation. Micro-CT imaging (Figure 8B) revealed that the initial bone tunnels were progressively occupied by dense trabecular bone; by 156 weeks, the voids previously occupied by the screws were nearly entirely replaced by host bone, indicating a synchronized process of material resorption and new bone formation. Histologically, the articular cartilage and joint capsule (Figure 8C) maintained normal morphology throughout the 3-year period, suggesting that the long-term release of degradation byproducts did not induce chronic inflammation or chondrocyte damage. High-magnification interface sections (Figure 8D) further highlighted active osseointegration, where newly formed bone trabeculae integrated seamlessly with the residual material. These findings demonstrate that the developed screw can be fully replaced by autologous bone within 156 weeks while maintaining excellent long-term biocompatibility and joint health.

**Figure 8:**
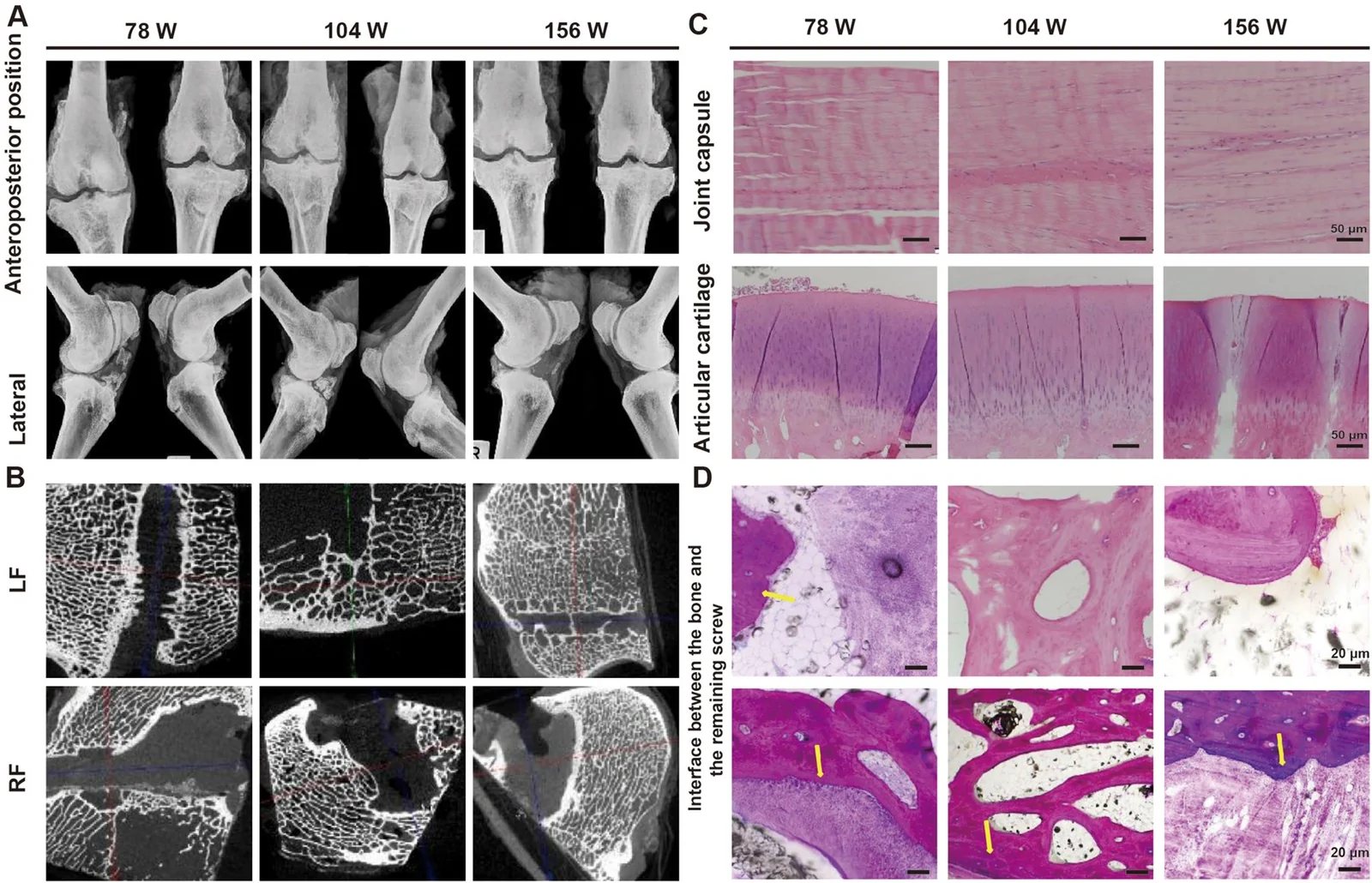
Long-term *in vivo* evaluation (78–156 weeks) of the absorbable interference screw. (**A**) Representative radiographs (AP and lateral) showing joint stability and absence of osteolysis. (**B**) Micro-CT images of LF and RF bone tunnels illustrating progressive bone infilling and tunnel narrowing. (**C**) H&E staining of the joint capsule and articular cartilage. (**D**) High-magnification images of the bone-screw interface. Arrows indicate new bone integration with the degrading material.

### The application of absorbable interference screws in the human body

To evaluate the efficacy and safety of the bioabsorbable interference screw system for the fixation of soft-tissue and BPTB grafts in orthopedic procedures, a multicenter clinical study was conducted using a single-arm target-value design. A total of 64 participants provided written informed consent and underwent orthopedic surgery using the bioabsorbable interference screw system. Clinical data were collected through a 6-month postoperative follow-up. The analysis populations included the Full Analysis Set (FAS), Per Protocol Set (PPS) and Safety Set (SS), as summarized in Table S4.

#### Primary efficacy endpoint

The primary efficacy endpoint was the proportion of participants achieving an excellent or good outcome according to the Lysholm Knee Scoring Scale at 6 months postoperatively.

In the FAS (Table 1), 60 of 63 participants (95.24%) achieved an excellent or good Lysholm rating at 6 months postoperatively. After adjustment for center effects, the rate of excellent or good outcomes was 96.79%, with a lower bound of the corresponding confidence interval of 91.78%, which exceeded the prespecified target value of 84.30%.

**Table 1:** Excellent and good rate of subjects at 6 months post-operation (FAS).

Item	Statistical description	Number of cases (Excellent + Good)	Total cases *n* (miss) = 63 (0)	Excellent and good rate (%)
Clinical efficacy	Center 1	20	22	90.91
	Center 2	23	24	95.83
	Center 3	17	17	100.00
	Excellent and good rate and 95% CI	95.24% (86.91, 98.37%)		
	Excellent and good rate and 95% CI adjusted for center effect	96.79% (91.78, 100.00%)		

Consistent results were observed in the PPS (Table 2). Among 59 participants, 56 (94.92%) achieved an excellent or good Lysholm rating at 6 months. After adjustment for center effects, the corresponding rate was 96.56%, with a lower confidence bound of 91.22%, also exceeding the prespecified target value of 84.30%.

**Table 2:** Excellent and good rate of subjects at 6 months post-operation (PPS).

Item	Statistical description	Number of cases (Excellent + Good)	Total cases *n* (miss) = 59 (0)	Excellent and good rate (%)
Clinical efficacy	Center 1	19	21	90.48
	Center 2	21	22	95.45
	Center 3	16	16	100.00
	Excellent and good rate and 95% CI	94.92% (86.08, 98.26%)		
	Excellent and good rate and 95% CI adjusted for center effect	96.56% (91.22, 100.00%)		

#### Secondary efficacy endpoints

The secondary efficacy endpoints included postoperative Lysholm scores and Lachman test results at 1, 3 and 6 months (Tables S5–S10).

At 1 month postoperatively, the mean Lysholm score in the FAS was 67.03 ± 16.68 points (range, 15–100 points). Two participants (3.28%) were rated as excellent, 5 (8.20%) as good, 28 (45.90%) as fair and 26 (42.62%) as poor. In the PPS, the mean score was 66.31 ± 16.45 points (range, 15–100 points), with 2 participants (3.39%) rated as excellent, 4 (6.78%) as good, 27 (45.76%) as fair and 26 (44.07%) as poor.

At 3 months postoperatively, the mean Lysholm score increased to 85.68 ± 13.70 points (range, 32–100 points) in the FAS. Twenty participants (31.75%) were rated as excellent, 23 (36.51%) as good, 14 (22.22%) as fair and 6 (9.52%) as poor. In the PPS, the mean score was 84.97 ± 13.80 points (range, 32–100 points), with 17 participants (28.81%) rated as excellent, 22 (37.29%) as good, 14 (23.73%) as fair and 6 (10.17%) as poor.

At 6 months postoperatively, further improvement in Lysholm scores was observed. In the FAS, the mean score reached 94.48 ± 6.12 points (range, 70–100 points), with 40 participants (63.49%) rated as excellent, 20 (31.75%) as good, 3 (4.76%) as fair and none (0.00%) as poor. In the PPS, the mean score was 94.29 ± 6.24 points (range, 70–100 points), with 37 participants (62.71%) rated as excellent, 19 (32.20%) as good, 3 (5.08%) as fair and none (0.00%) as poor.

Overall, the Lysholm scores showed a progressive improvement from 1 to 6 months postoperatively, accompanied by an increasing proportion of participants achieving excellent or good functional outcomes.

#### The Lachman test

At 1 month postoperatively (Tables S11 and S12), the Lachman test endpoint was firm in 60 participants (98.36%) and soft in 1 participant (1.64%) in the FAS, with no participants showing an absent endpoint. The Lachman test was negative in all 61 participants (100.00%). In the PPS, 58 participants (98.31%) had a firm endpoint and 1 (1.69%) had a soft endpoint; no absent endpoints were observed. All 59 participants (100.00%) had a negative Lachman test.

At 3 months postoperatively (Tables S13 and S14), all 63 participants in the FAS (100.00%) had a firm Lachman endpoint, with no soft or absent endpoints. The Lachman test was negative in all participants (100.00%). Similarly, in the PPS, all 59 participants (100.00%) had a firm endpoint and a negative Lachman test.

At 6 months postoperatively (Tables S15 and S16), the Lachman test results remained stable. In both the FAS and PPS, all participants had a firm endpoint (63/63 [100.00%] and 59/59 [100.00%], respectively), with no soft or absent endpoints. The Lachman test was negative in all participants in both analysis sets (100.00%).

Taken together, the clinical outcomes demonstrated progressive improvement in knee function, as reflected by the Lysholm scores, from 1 to 6 months after surgery. Meanwhile, the consistently negative Lachman test results and restoration of a firm endpoint indicated satisfactory postoperative anterior knee stability throughout the follow-up period.

## Conclusion

In conclusion, the 30% β-TCP/70% PLGA composite interference screw demonstrated favorable biocompatibility, mechanical stability and osteointegration potential in the present preclinical evaluations. The *in vivo* findings indicated gradual material degradation accompanied by new bone formation at the implant–bone interface, without evident adverse inflammatory responses during the observation period. These results suggest that the β-TCP/PLGA composite has potential as a bioabsorbable fixation material for ACL reconstruction while providing adequate initial mechanical support and a favorable environment for bone–implant integration. The retrospective clinical evaluation further provided preliminary evidence of its clinical feasibility and short-term safety; however, the single-arm study design, limited sample size and relatively short follow-up period preclude definitive conclusions regarding comparative clinical efficacy, long-term degradation behavior or long-term safety. Therefore, well-designed prospective controlled clinical studies with larger cohorts and extended follow-up are warranted to further establish the clinical effectiveness, degradation characteristics and long-term safety of this bioabsorbable interference screw.

## Supplementary Material

rbag211_Supplementary_Data
