# Supplementary material for: The application of absorbable interference screws in anterior cruciate ligament reconstruction of the knee joint
Source: Regen Biomater. 2026 Sep 24;13:rbag211. doi: 10.1093/rb/rbag211 (PMC13763427; doi:10.1093/rb/rbag211)
Supplement: Supplementary file 1 [file rbag211_supplementary_data.docx]

**Supplementary Materials**

**The application of absorbable interference screws in anterior cruciate ligament reconstruction of the knee joint**

Junfeng Li^a,b^, Jingying Dai^a^, Zhixiong Lin^b^, He yang^a^, Daimei Li^c^, Xiaoyan Chen^c^, Xinfeng Wang^b^, Jingru Du^d,*^, Honglian Dai^a,*^

a State Key Laboratory of Advanced Technology for Materials Synthesis and Processing, Hubei Key Discipline Laboratory of Orthopedic Tissue Injury and Repair, Wuhan University of Technology, Wuhan 430070, China

b The Third Hospital of Zhangzhou, Zhangzhou 363003, China

c Key Laboratory of Carbon Fiber Development and Application, School of Chemistry and Life Sciences, Changchun University of Technology, Changchun 130012, Jilin Province, China

d Department of Cardiology, The Second Affiliated Hospital of Fujian Medical University, Quanzhou 362000, China

* Corresponding authors

Junfeng Li, Zhixiong Lin and Jingying Dai contributed equally to this work.

E-mail: daihonglian@whut.edu.cn

Figure S1. (A) Characterization of absorbable interference screws using a stereoscopic zoom optical microscope. (B) Time schedule for ACL reconstruction of the knee joint in sheep. (C) Images of the surgical skin incision site or subcutaneous layer, as well as images of the distal organs of the animal at different weeks.

Table S1 Demographic data of the participants（FAS）

| Item | Descriptive statistics | Total |
| --- | --- | --- |
| Age（years） | n(miss) | 63 (0) |
|  | Mean±SD | 34.07±10.27 |
|  | Median(Q1-Q3) | 32.68(26.82-39.55) |
|  | Min-Max | 18.80-68.40 |
| Gender（cases） | n(miss) | 63 (0) |
|  | Male | 45(71.43) |
|  | Female | 18 (28.57) |
| Height（cm） | n(miss) | 63 (0) |
|  | Mean±SD | 173.04±8.59 |
|  | Median(Q1-Q3) | 173.00 (168.00-180.00) |
|  | Min-Max | 155.00-194.00 |
| Weight（kg） | n(miss) | 63 (0) |
|  | Mean±SD | 76.18±11.23 |
|  | Median(Q1-Q3) | 75.00 (68.40-82.00) |
|  | Min-Max | 52.00-102.00 |

Table S2 Baseline Lysholm Scale scores for participants (FAS)

| Item | Descriptive statistics | Total |
| --- | --- | --- |
| Lysholm score | n(miss) | 63 (0) |
|  | Mean±SD | 64.51±20.32 |
|  | Median(Q1-Q3) | 66.00(48.00-81.00) |
|  | Min-Max | 9.00-100.00 |
| Lysholm class | n(miss) | 63 (0) |
|  | Excellent | 3(4.76) |
|  | Good | 10(15.87) |
|  | Fair | 23(36.51) |
|  | Bad | 27 (42.86) |

Table S3 Baseline Lachman test results for subjects (FAS)

| Item | Descriptive statistics | Total |
| --- | --- | --- |
| Type of injured ligament | n(miss) | 63 (0) |
|  | Anterior cruciate ligament | 63(100.00) |
|  | Posterior cruciate ligament | 0 (0.00) |
| Lachman test endpoint | n(miss) | 63 (0) |
|  | Firm | 1(1.59) |
|  | Soft | 47 (74.60) |
|  | Absent | 15 (23.81) |
| Lachman test result | n(miss) | 63 (0) |
|  | Positive | 62 (98.41) |
|  | Negative | 1 (1.59) |

Table S4 Distribution of the cases enrolled in the subjects

| Description | Center01 | Center02 | Center03 | Total |
| --- | --- | --- | --- | --- |
| Enter the group | 22 | 25 | 17 | 64 |
| Drop | 0 | 0 | 0 | 0 |
| Reject | 0 | 1 | 0 | 1 |
| FAS | 22 | 24 | 17 | 63 |
| PPS | 21 | 22 | 16 | 59 |
| SS | 22 | 25 | 17 | 64 |

Table S5. 1-Month post-operative Lysholm scale results (FAS)

| Item | Descriptive statistics | Total |
| --- | --- | --- |
| Lysholm score | n(miss) | 61 (2) |
|  | Mean±SD | 67.03±16.68 |
|  | Median(Q1-Q3) | 67.00(56.00-80.00) |
|  | Min-Max | 15.00-100.00 |
| Lysholm class | n(miss) | 61 (2) |
|  | Excellent | 2(3.28) |
|  | Good | 5(8.20) |
|  | Fair | 28(45.90) |
|  | Bad | 26 (42.62) |

Table S6. 1-Month post-operative Lysholm scale results (PPS)

| Item | Descriptive statistics | Total |
| --- | --- | --- |
| Lysholm score | n(miss) | 59 (0) |
|  | Mean±SD | 66.31±16.45 |
|  | Median(Q1-Q3) | 67.00(55.00-80.00) |
|  | Min-Max | 15.00-100.00 |
| Lysholm class | n(miss) | 59 (0) |
|  | Excellent | 2(3.39) |
|  | Good | 4(6.78) |
|  | Fair | 27(45.76) |
|  | Bad | 26 (44.07) |

Table S7. 3-Month post-operative Lysholm scale results (FAS)

| Item | Descriptive statistics | Total |
| --- | --- | --- |
| Lysholm score | n(miss) | 63 (0) |
|  | Mean±SD | 85.68±13.70 |
|  | Median(Q1-Q3) | 90.00(84.00-95.00) |
|  | Min-Max | 32.00-100.00 |
| Lysholm class | n(miss) | 61 (0) |
|  | Excellent | 20(31.75) |
|  | Good | 23(36.51) |
|  | Fair | 14(22.22) |
|  | Bad | 6 (9.52) |

Table S8. 1-Month post-operative Lysholm scale results (PPS)

| Item | Descriptive statistics | Total |
| --- | --- | --- |
| Lysholm score | n(miss) | 59 (0) |
|  | Mean±SD | 84.97±13.80 |
|  | Median(Q1-Q3) | 89.00(80.00-95.00) |
|  | Min-Max | 32.00-100.00 |
| Lysholm class | n(miss) | 59 (0) |
|  | Excellent | 17(28.81) |
|  | Good | 22(37.29) |
|  | Fair | 14(23.73) |
|  | Bad | 6 (10.17) |

Table S9. 6-Month post-operative Lysholm scale results (FAS)

| Item | Descriptive statistics | Total |
| --- | --- | --- |
| Lysholm score | n(miss) | 63 (0) |
|  | Mean±SD | 94.48±6.12 |
|  | Median(Q1-Q3) | 95.00(94.00-100.00) |
|  | Min-Max | 70.00-100.00 |
| Lysholm class | n(miss) | 63 (0) |
|  | Excellent | 40(63.49) |
|  | Good | 20(31.75) |
|  | Fair | 3(4.76) |
|  | Bad | 0 (0.00) |

Table S10. 6-Month post-operative Lysholm scale results (PPS)

| Item | Descriptive statistics | Total |
| --- | --- | --- |
| Lysholm score | n(miss) | 59(0) |
|  | Mean±SD | 94.29±6.24 |
|  | Median(Q1-Q3) | 95.00(94.00-100.00) |
|  | Min-Max | 70.00-100.00 |
| Lysholm class | n(miss) | 59 (0) |
|  | Excellent | 37(62.71) |
|  | Good | 19(32.20) |
|  | Fair | 3(5.08) |
|  | Bad | 0 (0.00) |

Table S11. 1-Month Post-operative Lachman Test Assessment (FAS)

| Item | Descriptive statistics | Total |
| --- | --- | --- |
| Lachman test endpoint | n(miss) | 61 (2) |
|  | Firm | 60(98.36) |
|  | Soft | 1 (1.64) |
|  | Absent | 0 (0.00) |
| Lachman test result | n(miss) | 61 (2) |
|  | Positive | 0 (0.00) |
|  | Negative | 61 (100.00) |

Table S12. 1-Month Post-operative Lachman Test Assessment (PPS)

| Item | Descriptive statistics | Total |
| --- | --- | --- |
| Lachman test endpoint | n(miss) | 59 (0) |
|  | Firm | 58(98.31) |
|  | Soft | 1 (1.69) |
|  | Absent | 0 (0.00) |
| Lachman test result | n(miss) | 59 (0) |
|  | Positive | 0 (0.00) |
|  | Negative | 59 (100.00) |

Table S13. 3-Month Post-operative Lachman Test Assessment (FAS)

| Item | Descriptive statistics | Total |
| --- | --- | --- |
| Lachman test endpoint | n(miss) | 63 (0) |
|  | Firm | 63(100.00) |
|  | Soft | 0 (0.00) |
|  | Absent | 0 (0.00) |
| Lachman test result | n(miss) | 63 (0) |
|  | Positive | 0 (0.00) |
|  | Negative | 63 (100.00) |

Table S14. 3-Month Post-operative Lachman Test Assessment (PPS)

| Item | Descriptive statistics | Total |
| --- | --- | --- |
| Lachman test endpoint | n(miss) | 59 (0) |
|  | Firm | 59(100.00) |
|  | Soft | 0 (0.00) |
|  | Absent | 0 (0.00) |
| Lachman test result | n(miss) | 59 (0) |
|  | Positive | 0 (0.00) |
|  | Negative | 59 (100.00) |

Table S15. 6-Month Post-operative Lachman Test Assessment (FAS)

| Item | Descriptive statistics | Total |
| --- | --- | --- |
| Lachman test endpoint | n(miss) | 63 (0) |
|  | Firm | 63(100.00) |
|  | Soft | 0 (0.00) |
|  | Absent | 0 (0.00) |
| Lachman test result | n(miss) | 63 (0) |
|  | Positive | 0 (0.00) |
|  | Negative | 63 (100.00) |

Table S16. 6-Month Post-operative Lachman Test Assessment (PPS)

| Item | Descriptive statistics | Total |
| --- | --- | --- |
| Lachman test endpoint | n(miss) | 59 (0) |
|  | Firm | 59(100.00) |
|  | Soft | 0 (0.00) |
|  | Absent | 0 (0.00) |
| Lachman test result | n(miss) | 59 (0) |
|  | Positive | 0 (0.00) |
|  | Negative | 59 (100.00) |
